# Heterogeneous Multi-Phase Grains Improving the Strength-Ductility Balance in Warm-Rolled Fe-18Mn-3Ti Steel

**DOI:** 10.3390/ma17112590

**Published:** 2024-05-28

**Authors:** Yifeng Li, Shulin Liu, Yuanguang Xia, Juping Xu, Huaican Chen, Wen Yin

**Affiliations:** 1 Institute of High Energy Physics, Chinese Academy of Sciences (CAS), Beijing 100049, China; yfli@ihep.ac.cn (Y.L.); slliu@ihep.ac.cn (S.L.); xiayg@ihep.ac.cn (Y.X.); xujuping@ihep.ac.cn (J.X.); chenhuaican@ihep.ac.cn (H.C.); 2 Spallation Neutron Source Science Center, Dongguan 523803, China; 3 School of Nuclear Science and Technology, University of Chinese Academy of Sciences, Beijing 100049, China

**Keywords:** high manganese steel, strength-ductility balance, warm rolling process, heterogeneous microstructure

## Abstract

The thermal properties, microstructure, and mechanical properties of Fe-18Mn-3Ti (wt%) were investigated, focusing on the effects of different heat-treatment processes. Results revealed that the 450 °C warm-rolling sample (450 WR) exhibited promising mechanical properties. Specifically, this sample displayed a yield strength of 988 MPa, an ultimate tensile strength of 1052 MPa, and total elongation of 15.49%. Consequently, a favorable strength-ductility balance was achieved. The strain-hardening ability surpassed that of the cold rolling sample (CR). Microstructure analysis indicated the simultaneous occurrence of dynamic equilibrium between grain deformation and re-crystallization because of the co-influence of thermal and strain in the warm rolling process. This desirable mechanical property was attributed to the presence of a multi-phase (α-martensite, austenite, and ε-martensite) and heterogeneous microstructure. The improvement of ultimate tensile strength was based on grain refinement, grain co-deformation, and the transformation-induced plasticity (TRIP) effect in the early stage of plastic deformation (stage Ⅰ). The improvement of ultimate elongation (TEL) was ascribed to the TRIP effect in the middle stage of plastic deformation (stage Ⅱ).

## 1. Introduction

In the past few decades, high manganese (high-Mn) steels have received significant attention in the automotive industry due to their excellent mechanical properties [1]. These high-Mn steels, typically consisting of 18–31 wt% Mn, have garnered significant attention in industrial production due to the high total energy of tensile (ultimate tensile strength × total elongation, UTS × TEL), low-temperature toughness, wear resistance, and non-magnetic properties [1,2,3]. It can be widely used in Gas-insulated switches, automobiles, and LNG storage tanks [4].

The second-generation advanced high-strength steels (2nd AHSS), mainly including high-Mn twinning-induced plasticity (TWIP) steels and high-Mn TRIP steels, possesses outstanding strength and remarkable ductility [2]. The improvement of ductility is mainly based on the TWIP or TRIP effect [1,5,6], and the improvement of strength is mainly ascribed to the formation of precipitates [4,7] or grain refinement [8,9]. Numerous studies have demonstrated that the addition of alloying elements such as Mo, Nb, and V can enhance their strength or ductility [10,11,12]. However, the high cost associated with these elements hampers the widespread application of high-Mn steels in industrial processes [2]. Meanwhile, Ti, one of the alloying elements, can bring exceptional mechanical properties to high-Mn steels with reasonable raw material costs (compared with Mo, Nb, V) [4,13,14]. 

The Ti plays an important role in the mechanical properties of manganese steels, especially in the improvement of yield strength of manganese steels. However, excessive Ti element can lead to a significant decrease in ductility, which leads to a decline in the total energy of tensile [15]. Hence, studies focus on searching for the appropriate titanium content to achieve an improvement in the strength and ductility of high-Mn steels [4,14]. Hu et al. reported that the addition of trace Ti (0.032 wt%) can increase the density of nanoscale TiC precipitates and refined austenite grains by inter-critical annealing treatment. Following this method, a Ti-alloyed Mn-steel with an ultrahigh tensile strength of 790 MPa and elongation of 27.4% is achieved [14]. Recently, a sustainable ultrahigh-strength Fe-18Mn-3Ti steel containing α-Mn nanoprecipitation was achieved, which possesses an ultrahigh tensile strength of 1.8 GPa and a ductility of 7% [4]. Meanwhile, this Fe-18Mn-3Ti steel also displayed high dynamic toughness (88 ± 12 MPa√m). According to the above studies, obtaining a favorable strength-ductility balance in Ti-containing manganese steels by tuning Ti content remains a challenge, especially incorporating low-cost alloyed elements. 

Recently, an excellent balance between strength and ductility has been achieved in Mn steels through various techniques, for example, warm rolling, hetero-deformation induced (HDI) stress, and ultrafast heating combined with inter-critical annealing [5,16,17,18,19]. The warm rolling process completely conforms to the existing production process conditions and is an effective method to achieve the combination of ultrahigh strength and excellent plasticity. This process is a breakthrough in the performance of Mn steels. For instance, two-stage warm rolling (TWR) processes have yielded dual-phase microstructures (α-martensite and austenite) [5] or multiple morphologies (lath-type and granular) microstructures [19], which effectively maintain the balance between strength and ductility in Mn steels. Moreover, an inter-critical rolling process without annealing could improve the fraction and stability of retained austenite, which improves strength and ductility simultaneously due to the persistent TRIP effect [6].

Here, a strength-ductility balance in high-Mn steel, Fe-18Mn-3Ti (wt%), was achieved by warm rolling method. The thermal properties, phase composition, microstructure, and mechanical properties of the Fe-18Mn-3Ti were investigated at different stages. Through a two-stage rolling process (hot rolled at 1100 °C and warm rolled at 450 °C), we successfully achieved a yield strength of 988 MPa, an ultimate tensile strength of 1052 MPa, and a total elongation of 15.49% in Fe-18Mn-3Ti. This two-stage rolling process can optimize material performance (strength-ductility balance) and reduce the reliance on expensive alloying elements, which has the advantage of economy and practicality in automotive and structural engineering.

## 2. Experiment Procedure

**Figure 1:**
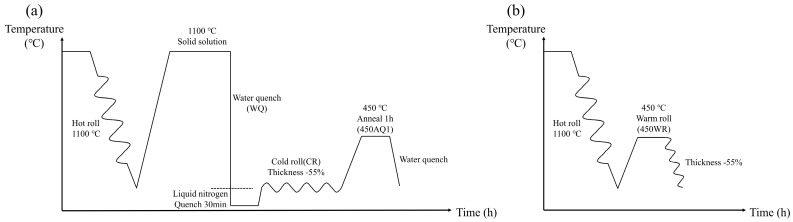
The flow chart of different heat-treatment processes in Fe-18Mn-3Ti steels. (**a**) The flow chart of WQ, CR, 450 AQ1 samples. (**b**) The flow chart of 450 WR samples.

The Fe-18Mn-3Ti steel utilized in this study was produced using a vacuum induction furnace and cast into rectangular sample billets measuring 100 mm (length) × 100 mm (width) × 30 mm (thickness). In the casting process, the mold material is made of high-purity elemental metal to ensure the high cleanliness of Fe-18Mn-3Ti steel. Table 1 presents the theoretical and experimental chemical compositions of the designed Fe-18Mn-3Ti steel obtained through X-ray fluorescence (XRF) and inductively coupled plasma emission spectrometry (ICP). The Carbon content is close to 0.014%, as measured by the Carbon and Sulfur Analyzer (Leco CS844, Leco Corporation, St. Joseph, MI, USA). The billet was subsequently hot-rolled at 1100 °C, which reduced its thickness from 30 mm to 6 mm in three passes at 40% strain per pass. Following this process, the plate was divided into sheets measuring 70 mm (length) × 30 mm (width) × 6 mm (thickness), forming four groups with distinct heat-treatment processes as outlined in Figure 1. One of the groups underwent annealing at 450 °C for 1 h, followed by warm rolling to reduce its thickness from 6 mm to 2.7 mm in 10 passes at 7.7% strain per pass (denoted by 450 WR) (Figure 1b). 

Three additional groups were extracted from the plate and subjected to solution treatment at 1100 °C for 3 h. Among these, one group was water quenched (denoted by WQ). The other two groups were liquid N_2_ quenched for 30 min and then underwent cold rolling (denoted by CR) to achieve a thickness reduction of 55%. Finally, one of the groups was annealed at 450 °C for 1 h and was water quenched (denoted by 450 AQ1). The above heat-treatment processes are shown in Figure 1a. The PanPhaseDiagram module in the Pandat 2022 software was used to evaluate the equilibrium phase fraction of the Fe-18Mn-3Ti steels [20]. The PanFe 2022b database was used to obtain the thermodynamic information of the Fe-18Mn-3Ti steels.

The austenite transformation temperature was measured by differential scanning calorimetry (DSC, STA449F3, NETZSCH, Tirschenreuth, Selb, Germany). The dimensions of DSC samples were 3 mm (length) × 2 mm (width) × 1 mm (thickness). During DSC measurement, the samples were placed in alumina pans and protected by a high-purity argon atmosphere with a gas flow speed of 20 mL/min. For thermodynamic studies, samples were heated from room temperature to 1000 °C with a heating rate of 10 °C/min, held for 5 min at 1000 °C, and cooled to room temperature.

The neutron diffraction pattern (NDP) was obtained by a 360° rotation stage in a Multi-Physics Instrument (MPI) at China Spallation Neutron Source (CSNS) [21]. The power of the neutron source was 140 kW. The time-of-flight (TOF) method was used to collect data for each sample over a total scanning time of 30 min. The NDP of different samples (before and after the tensile test) were analyzed by modified Rietveld refinement software GSAS II (SVN version 5258) [22] to determine the volume fractions of α-martensite, austenite, and ε-martensite. For microstructure examination, the investigated samples were analyzed by using scanning electron microscopy (SEM, ZEISS Sigma 500, Jena Zeiss, Jena, Germany) equipped with an instrument for electron backscatter diffractometry (EBSD Hikari Plus). The EBSD data were analyzed by using the software Channel5 (5.0.9.0) to identify the volume fraction of phases and the distribution of local average misorientation (LAM).

Tensile tests were performed on a Suns electronic universal testing machine. To ensure reliability, three tensile specimens for each heat-treatment processes with a gauge size of 2 mm × 4 mm × 15 mm were machined along the rolling direction and tested at a strain rate of 10^−3^ s^−1^.

## 3. Results and Discussion

### 3.1. Mechanical Properties

**Figure 2:**
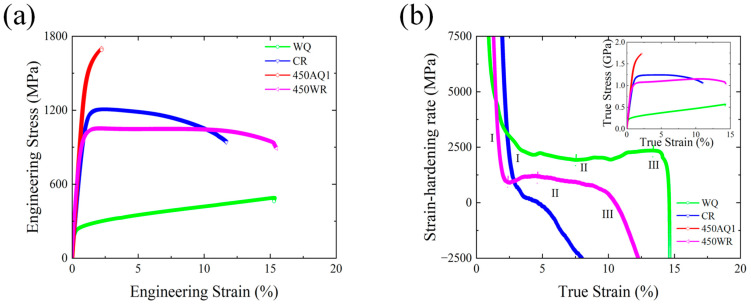
Mechanical properties of different samples subjected to various processes. (**a**) Engineering stress-strain curves. (**b**) True stress-strain hardening rate curves. The inset is the true stress-strain curves.

Figure 2a displays the engineering stress-strain curves of different samples. Figure 2b represents the strain-hardening ability of different samples. For the WQ and 450 WR samples, the plastic deformation can be divided into three stages. The WQ sample exhibited an UTS of 489.41 GPa (Figure 2a) and a TEL of 15.34% (Figure 2a). After the cold rolling process, the strength is significantly improved. However, due to the decline of elongation, the strain-hardening process of the CR sample is suppressed (Figure 2b). The UTS of the CR sample reached 1211 MPa, while the TEL dropped to 11.68%. After the annealing and quenching process, the 450 AQ1 sample fractured before reaching the UTS (TEL = 2.2%), which led to the brittle fracture. Thus, the CR and 450 AQ1 samples have been restricted by a strength-ductility balance. The UTS × TEL of 450 WR sample exhibits 16.3 GPa% (Table 2), which is higher than other experimental steels [23,24,25]. The 450 WR sample achieves the desired strength and ductility balance. In addition, the 450 WR sample shows a better strain-hardening ability than the CR sample (Figure 2b). Although Silva et al. found that the 450 ℃ aging process can significantly improve the UTS of Fe-18Mn-3Ti (1838 MPa after the 3 h aging process at 450 °C), the TEL of Fe-18Mn-3Ti decreases to 7% [4], which leads to the decline of UTS × TEL (12.9 GPa%). 

### 3.2. Thermal Properties

**Figure 3:**
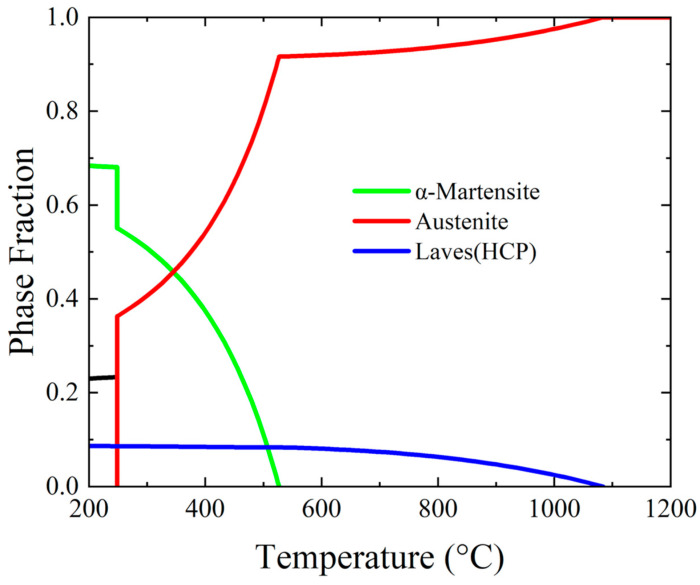
Equilibrium phase diagram of Fe-18Mn-3Ti.

Figure 3 illustrates the phase fraction of Fe-18Mn-3Ti determined through thermodynamic equilibrium analysis using the Pandat2022 software [20]. The phase fraction assessment for Fe-18Mn-3Ti at 1100 °C reveals complete solidification into austenite. At 450 °C, the samples predominantly consist of austenite, α-martensite, and 8% Laves (Fe_2_Ti) phases (with hexagonal lattice structure). At 520 °C, the α-martensite was completely transformed into austenite. Although the database predicts that the Laves phase is an equilibrium phase at 450 °C, the multi-step nucleation pathway and the high energy of the incoherent interface suppress the formation of the Laves phase [4]. 

**Figure 4:**
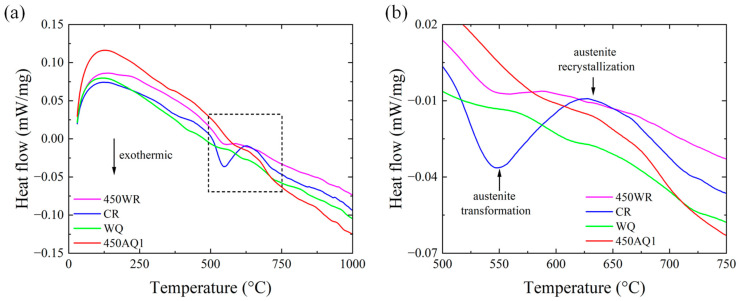
(**a**) The DSC curves of different samples. (**b**) The enlarged DSC curves. The austenite transformation and recrystallization temperature are shown in DSC curves.

Figure 4 shows the DSC curves measured during continuous heating. Table 3 shows the austenite transformation and recrystallization temperature of different samples. Except for the WQ sample, the exothermic peak can be observed in the other three samples. The austenite transformation temperature of CR and 450 WR samples are in good agreement with the prediction of the equilibrium phase diagram (Figure 3). The exothermic peak intensity of 450 WR samples is significantly lower than CR samples, indicating that the phase fraction of α-martensite decreases in the warm rolling process. The 450 AQ1 sample exhibits higher austenite transformation and recrystallization temperature than the other two samples, which indicates that the annealing process at 450 °C enhances the stacking fault probability [26]. These findings offer practical guidance for optimizing the heat treatment process of Fe-18Mn-3Ti austenitic steel.

### 3.3. Microstructure Analysis

**Figure 5:**
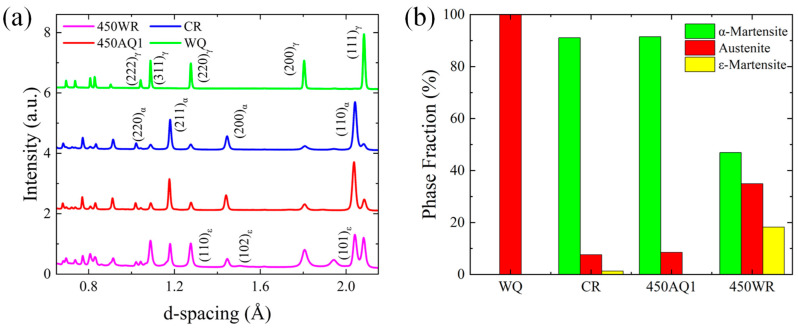
(**a**) The neutron diffraction pattern (NDP). (**b**) Histogram of phase fraction of different thermal treatment processing.

Figure 5 shows the NDP of the samples subjected to various thermal treatments. According to the thermodynamic equilibrium analysis in Figure 3, due to the high driving force of austenite transformation at 1100 °C, full austenite was formed in the WQ sample. The formation of the austenite in the WQ sample led to its low yield strength. After the rolling process at room temperature, the peak intensity of α-martensite rises (91%), which may lead to the improvement of yield strength and the decline of elongation. The 450 ℃ annealing process can restrict the formation of ε-martensite (with hexagonal lattice structure). Combined with Table 3, this temperature was lower than Ac_1_, so it is difficult to observe the austenite transformation. The phase fraction of retained austenite (8.5%) remained at a low level, which was significantly lower than the value reported in a reference study [4]. Compared with the CR sample, the warm rolling process led to the peak intensity of retained austenite (34.9%) and ε-martensite (18.2%) rising, which led to the improvement of elongation. The warm rolling process in 18 wt% Mn low carbon steels can improve the stability of ε-martensite because of the high driving force energy of ∆G^ε→α^ [27]. Combined with the DSC results (Figure 4), the 450 WR samples exhibited similar Ac_1_ and A_Rc to the CR samples, which reflected that the two stages of transformation (α→ε→γ) occurred in 450 WR samples. The two stages of transformation (γ→ε→α) mechanism in the cooling process can also be observed in Fe-12Mn steels [27], which indicates that strain-induced ε-martensite has low thermal stability, and it is more likely to transform into austenite.

Based on the thermal properties and the phase identification results, in the warm rolling process, the combination of thermal and deformation leads to the high stability of strain-induced α-martensite and ε-martensite at room temperature. Compared with the WQ samples, the formation of martensite (α and ε) can significantly improve the yield strength in the tensile test (Figure 2a).

**Figure 6:**
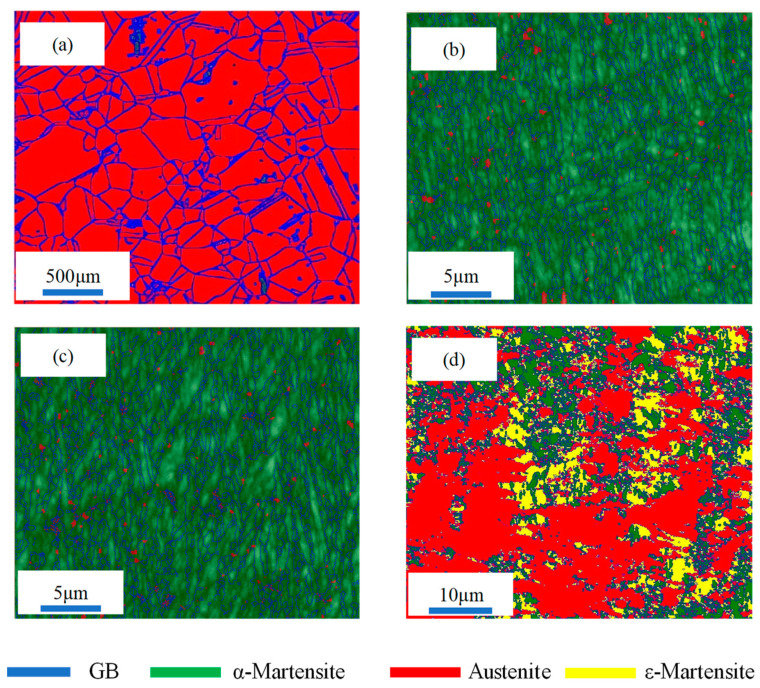
The phase distribution before tensile tests: (**a**) WQ, (**b**) CR, (**c**) 450 AQ1, (**d**) 450 WR, respectively.

**Figure 7:**
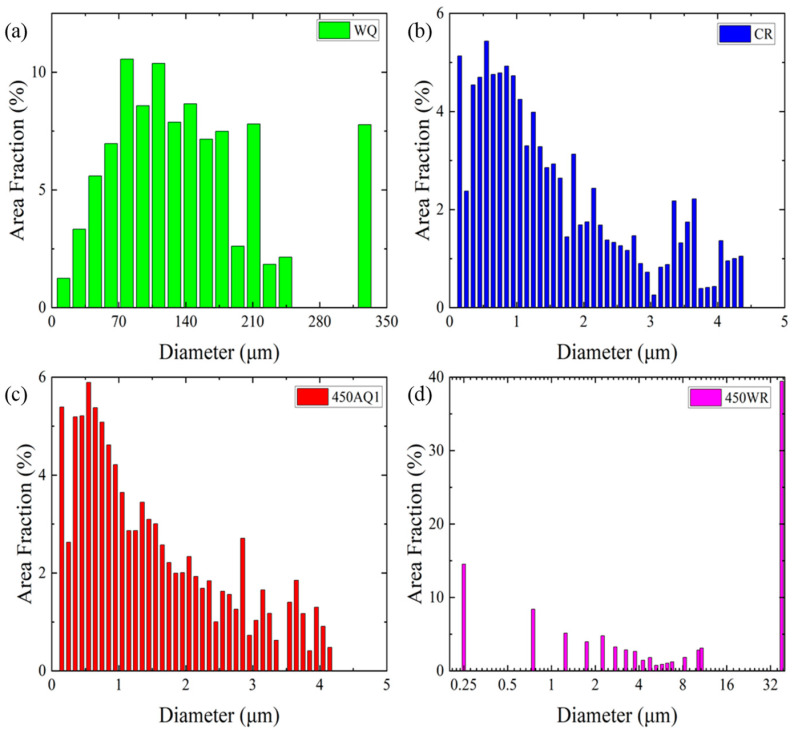
The grain size histogram before tensile tests: (**a**) WQ, (**b**) CR, (**c**) 450 AQ1, (**d**) 450 WR, respectively.

The grain size and phase distribution analysis of the different samples by EBSD are presented in Figure 6 and Figure 7. The WQ sample is composed of coarse austenite grains and exhibits an average grain size of 140 μm (Figure 7a), which leads to low yield strength. According to the Hall–Petch effect [28], the coarse grain size of the WQ sample can promote the movement of dislocations and decrease the strength of the WQ sample. After the rolling process, the CR, 450 AQ1, and 450 WR samples all present ultrafine grains. Lath α-martensite grains are primarily formed in the CR and 450 AQ1 samples. Meanwhile, a small amount of nano-sized austenite grains was observed at the grain boundaries of the lath α-martensite. Moreover, the average grain size of the 450 AQ1 sample (1.48 μm) was smaller than that of the CR samples (1.55 μm). This may result from the enhanced segregation of Mn to the dislocation during 450 °C annealing treatment, which increases nucleation sites at grain boundaries [29]. In Figure 6d, an inhomogeneous microstructure consisting of granular multi-phase grains (<2 μm) and lamellar austenite grains (from 2 μm to 40 μm) can be observed in the 450 WR sample. No twin phases were observed in Figure 6d. According to Yang et al.’s study, the granular grains with lower yield strength deform first, followed by lamellar grains with higher yield strength, and eventually undergo co-deformation [30]. Hence, these inhomogeneous grains may improve the strain-hardening ability of 450 WR sample.

**Figure 8:**
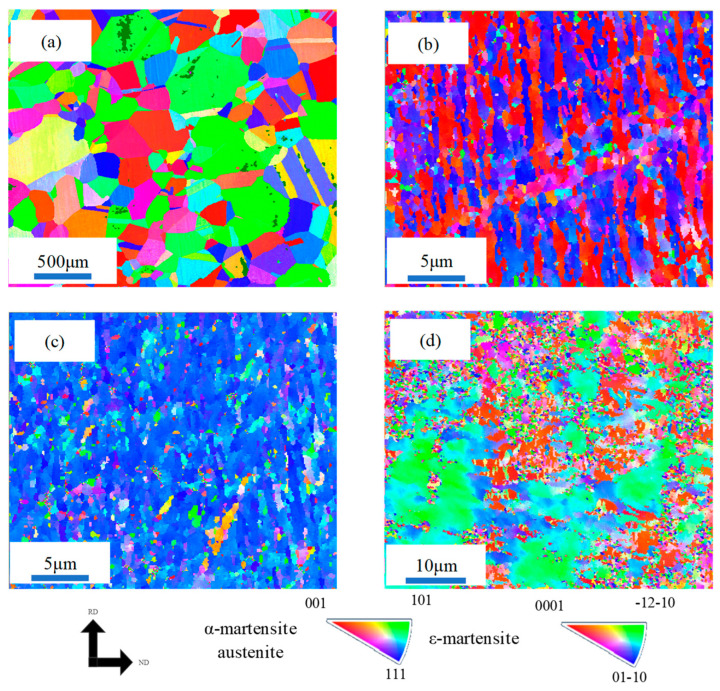
The orientation imaging maps (OIM) for all phases before tensile tests: (**a**) WQ, (**b**) CR, (**c**) 450 AQ1, (**d**) 450 WR, respectively.

**Figure 9:**
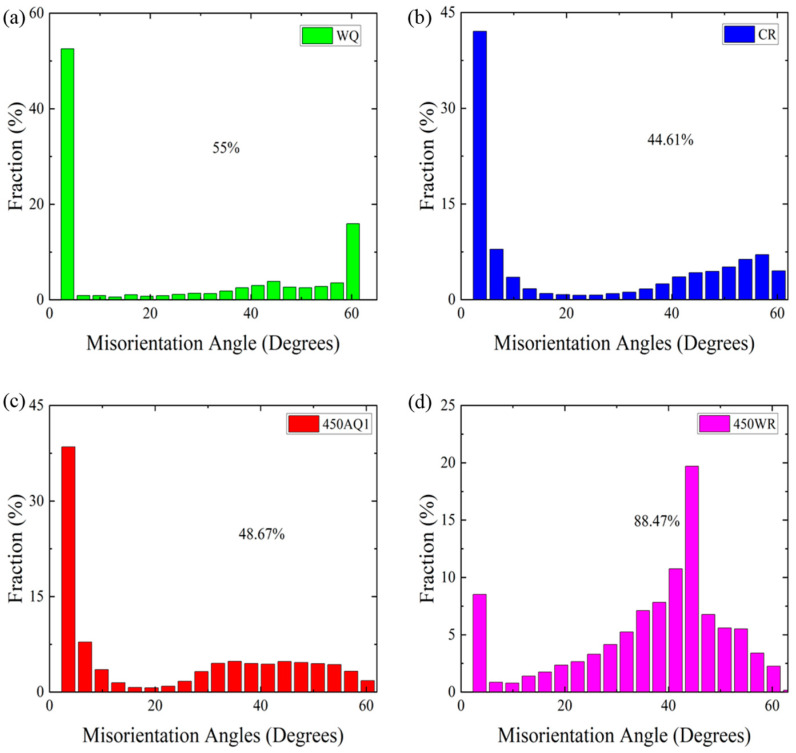
The misorientation histogram imaging for all phases before tensile tests: (**a**) WQ, (**b**) CR, (**c**) 450 AQ1, (**d**) 450 WR, respectively.

The OIM of the different samples is shown in Figure 8a–d. The <001> direction was primarily distributed in areas with high defect density, whereas the <111> direction was predominantly distributed in regions with low defect density (Figure 8c). This indicates that the cold-rolling process induces severe plastic deformation (SPD) [29]. The <111> direction of α-martensite grains in the 450 AQ1 sample tended to align parallel to the normal direction (ND) (Figure 8c). Interestingly, apparent anisotropy was observed in the 450 WR sample. The distribution of misorientation in the different samples is shown in Figure 9 a–d. The high grain misorientation (450 WR is 88.47%, WQ is 55%) may blunt and arrest the propagation of microcracks and improve the elongation [30,31].

**Figure 10:**
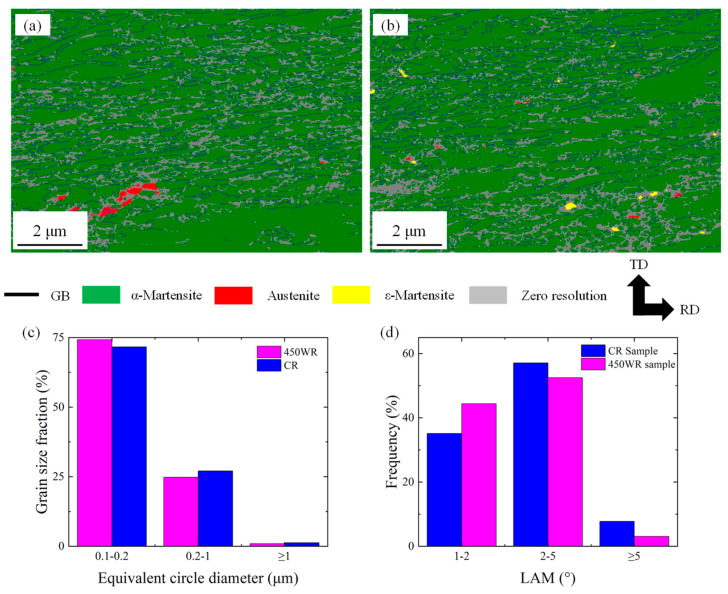
The microstructures of tensile samples near the fracture surface. The phase distribution of (**a**) CR and (**b**) 450 WR samples. The histogram of (**c**) grain size and (**d**) local misorientation (LAM) for different samples.

Figure 10a,b show the phase distribution for CR and 450 WR samples. The black line represents grain boundaries. The gray area represents the grain boundaries and microcracks due to the severe deformation of grains in the rolling direction. For the CR sample (Figure 10a), the deformation of lath α-martensite grains dominates the plastic deformation, which may restrict the strain hardening ability. Chen et al. reported that the refinement of grains is adverse to the stability of austenite and promotes the TRIP effect [32]. As such, the grain refinement effect is more obvious than in the CR sample (Figure 10c). The above results indicate that both the TRIP effect and grain refinement effect play an important role in strain hardening behaviors. Figure 10d shows that the LAM of the 450 WR sample is lower than the CR sample, indicating that the microcrack propagation in the 450 WR sample may be suppressed.

In addition, Li et al. found that for low-carbon high-Mn steels, with the refinement of austenite grains, the γ→ε (austenite transforms into ε-martensite) transformation is suppressed. The γ→α (austenite transforms into α-martensite) transformation in coarse austenite grains is more likely to occur [33]. Here, for the 450 WR sample (Figure 10b), combined with the microstructure before the tensile test Figure 6d, it indicates that the warm rolling process promotes the γ→α and γ→ε transformation. Moreover, the γ→α and ε→α (ε-martensite transforms into α-martensite) transformation are occurred during the tensile test.

**Figure 11:**
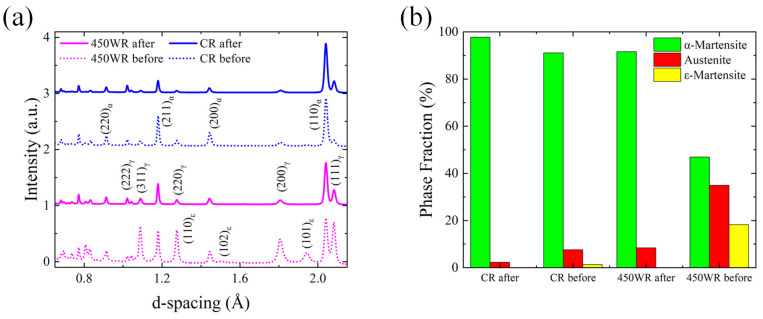
NDP results with CR and 450 WR samples before and after the tensile test (the whole deformation area in the tensile sample) (**a**) NDP patterns. (**b**) Phase fraction obtained by GSAS II.

The NDP results before and after the tensile test are shown in Figure 11. The peak intensity of retained austenite in both CR (2.3% of austenite) and 450 WR (8.4% of austenite) samples falls after the tensile strain, indicating the formation of strain-induced α-martensite. Combined with the EBSD results (Figure 6d and Figure 10b), the 450 WR sample shows a larger initial work hardening rate (Figure 2b) is credited to the unstable lamellar austenite grains. With the increasing strain level, the lamellar austenite grains refine gradually and transform into elongated α-martensite grains. Yang et al. [30] reported that the microstructural heterogeneity in the high specific strength steels led to grain co-deformation and high back stresses, which is beneficial for elongation.

The peaks of ε-martensite disappear, indicating that the ε-martensite transforms into α-martensite during the early stage of deformation. Li et al. reported that the γ→ε and ε→α collaborative transformation during stage Ⅱ of tensile deformation in 17Mn steel leads to the delay of necking, which offers high ultimate elongation [33]. The γ→α transformation during stage Ⅰ of tensile deformation in 15Mn steel offers a high strain hardening rate and improves the UTS [33]. Combined with the strain hardening curves (Figure 2b), it can be indicated that the γ→α transformation during stage Ⅰ improves the strain hardening capability. The γ→ε and ε→α collaborative transformation during stage Ⅱ improves the elongation of the 450 WR sample.

**Figure 12:**
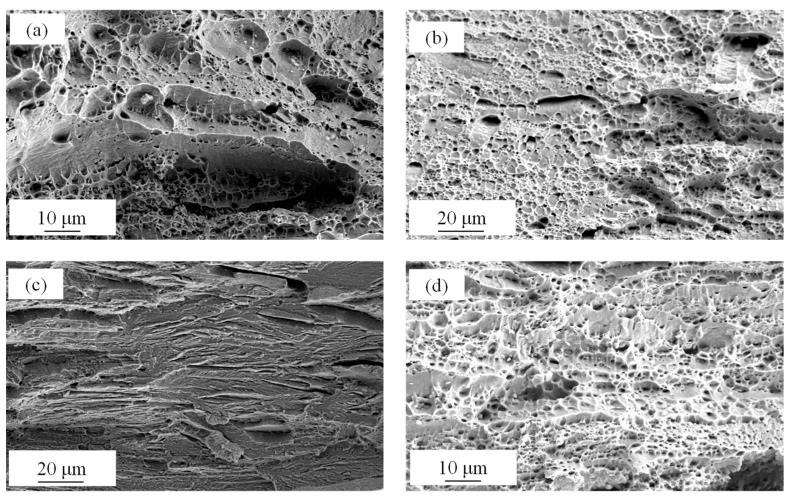
Fractography of different samples after tensile tests: (**a**) WQ, (**b**) CR, (**c**) 450 AQ1, (**d**) 450 WR.

Figure 12 shows the fracture surfaces of different samples subjected to various processes. The WQ sample (Figure 12a) shows coexisting oblique cleavage facets and large dimples. The CR sample (Figure 12b) shows shallow dimples and a high density of small cleavage facets, which is affected by the brittle phase of lath α-martensite. The 450 AQ1 sample exhibits numerous smooth cleavage surfaces and cleavage cracks (Figure 12c), which is characteristic of transgranular fracturing. Due to the TRIP effect in the tensile test and lower LAM (Figure 10d) (compared with the CR sample) near the fracture surface, the homogeneous dimples and few cleavage facets are obtained in the 450 WR sample (Figure 12d). It is advisable to avoid heavy cold-rolling in high-strength steels to prevent the formation of a high-density texture, which can lead to low ductility and severe brittle fracture.

## 4. Conclusions

In this study, a two-stage rolling process was applied to the Fe-18Mn-3Ti steel. The balance between strength and ductility, and the relationship between mechanical properties, thermal properties, and microstructure are discussed. The main conclusions are as follows:

(1) After the warm rolling process, the Fe-18Mn-3Ti steel achieves the strength and ductility balance with the ultimate tensile strength of 1052 MPa and a total elongation of 15.49%. In addition, the strain-hardening ability is significantly improved.
(2) The 450 AQ1 sample has higher austenite transformation temperature (Ac_1_: 580 °C, Ac_3_: 630 °C) than the 450 WR (Ac_1_: 503 °C, Ac_3_: 595 °C) and CR (Ac_1_: 506 °C, Ac_3_: 612 °C) samples. Besides, the ε-martensite is induced by strain and has low thermal stability, which leads to a two-stage phase transformation in the annealing process.
(3) The 450 WR sample consists of heterogeneous (containing lamellar and granular) austenite, granular α-martensite, and granular ε-martensite. The martensite transformation in the warm rolling process increases the yield strength. The heterogeneous microstructure leads to grain co-deformation and improves elongation. Besides, the high grain disorientation in the 450 WR sample enhances the strain-hardening ability.
(4) The persistent TRIP effect plays an important role in improving the mechanical properties. During stage Ⅰ of the tensile test, the lamellar austenite grains refine gradually and transform into elongated α-martensite grains. The grain refinement effect and the formation of strain-induced α-martensite improve the strain-hardening ability and ultimate tensile strength. During the stage Ⅱ of the tensile test, the γ→ε and ε→α collaborative transformation improves the elongation.

Notably, the adoption of a two-stage rolling process offers a simplified and cost-effective approach for producing high-Mn steels, which provides an economical scheme to apply in industrial production.

## Figures and Tables

**Table 1:** Chemical composition of Fe-18Mn-3Ti a steel in wt.%.

Composition (wt%)	Fe	Mn	Ti	Ni	Al	C
theory	Balance	18	3			
ICP	Balance	17.75	2.63	0.0068	0.0068	
XRF	Balance	17.39	2.49	0.009	0.012	0.006

**Table 2:** The total energy of tensile, UTS × TEL, for experimental reference data are compared with 450 WR samples.

Samples	UTS /MPa	TEL /%	UTS × TEL /GPa%
450 WR (this work)	1052	15.49	16.30
Fe-18Mn-3Ti [4]	1767	8.36	14.78
Ti-LWS [23]	767	9.97	7.65
Fe-9Mn [24]	821	6.76	5.55
Fe-0.51Mn-0.82Cr-0.61Ti-0.61Ni [25]	1484	9.90	14.69

**Table 3:** The austenite transformation temperature of different samples obtained by the DSC curves. The Ac_1_ (°C) and Ac_3_ (°C) represent the start and end of austenite transformation. The A_Rc (°C) represents the austenite recrystallization temperature.

Sample	Ac_1_ (°C)	Ac_3_ (°C)	A_Rc (°C)
CR	506	612	641
450 AQ1	580	630	656
450 WR	503	595	637

## Data Availability

Data are contained within the article.
